# Carbon Emissions in China’s Construction Industry: Calculations, Factors and Regions

**DOI:** 10.3390/ijerph15061220

**Published:** 2018-06-10

**Authors:** Qiang Du, Xinran Lu, Yi Li, Min Wu, Libiao Bai, Ming Yu

**Affiliations:** 1 School of Economics and Management, Chang’an University, Xi’an 710064, China; q.du@chd.edu.cn (Q.D.); liyi@chd.edu.cn (Y.L.); 2 School of Civil Engineering, Chang’an University, Xi’an 710061, China; lu_xinran@chd.edu.cn (X.L.); min_wu@chd.edu.cn (M.W.); ming_yu@chd.edu.cn (M.Y.)

**Keywords:** construction industry, carbon emissions, LMDI, province of China

## Abstract

The production of construction projects is carbon-intensive and interrelated to multiple other industries that provide related materials and services. Thus, the calculations of carbon emissions are relatively complex, and the consideration of other factors becomes necessary, especially in China, which has a massive land area and regions with greatly uneven development. To improve the accuracy of the calculations and illustrate the impacts of the various factors at the provincial level in the construction industry, this study separated carbon emissions into two categories, the direct category and the indirect category. The features of carbon emissions in this industry across 30 provinces in China were analysed, and the logarithmic mean Divisia index (LMDI) model was employed to decompose the major factors, including direct energy proportion, unit value energy consumption, value creation effect, indirect carbon intensity, and scale effect of output. It was concluded that carbon emissions increased, whereas carbon intensity decreased dramatically, and indirect emissions accounted for 90% to 95% of the total emissions from the majority of the provinces between 2005 and 2014. The carbon intensities were high in the underdeveloped western and central regions, especially in Shanxi, Inner-Mongolia and Qinghai, whereas they were low in the well-developed eastern and southern regions, represented by Beijing, Shanghai, Zhejiang and Guangdong. The value creation effect and indirect carbon intensity had significant negative effects on carbon emissions, whereas the scale effect of output was the primary factor creating emissions. The factors of direct energy proportion and unit value energy consumption had relatively limited, albeit varying, effects. Accordingly, this study reveals that the evolving trends of these factors vary in different provinces; therefore, overall, our research results and insights support government policy and decision maker’s decisions to minimize the carbon emissions in the construction industry.

## 1. Introduction

The issue of global climate change has attracted increasing attention in recent years, largely because of its serious consequences with respect to the natural and human environment [1]. Moreover, China, which became the world’s largest carbon emitter in 2006, was responsible for 64.8% of the increase in global carbon emissions between 2007 and 2012 [2]. In 2013, China emitted approximately 10.2 million kilo tons (kt) carbon emissions, which made up 28.6% of the world’s total carbon emissions and was nearly double that of the United States (5.2 million kt) [3]. As China is facing great pressure from the international community on the issue of carbon emissions, the government is making effect to control the carbon emissions. In 2014, China pledged, in the US-China Joint Announcement on Climate Change, that it would reach its peak of carbon emissions around 2030 [4].

The construction industry is one of the key industries contributing to carbon emissions in China [5]. The important role of the construction industry in the Chinese economy and the large amount of resources consumed by the industry have resulted in a substantial amount of carbon emissions. Chuai et al. [6] estimated that the carbon emissions from the construction industry made up 27.9% to 34.3% of the overall carbon emissions in China between 1995 and 2010. Reducing the construction industry carbon emissions can significantly help China reach its emissions reduction target. Moreover, the construction industry is a carbon-intensive industry that consumes a considerable amount of energy from other industries. Therefore, to provide a basis for Chinese policymakers to formulate appropriate policies to reduce the industry-wide carbon emissions, it is important to accurately calculate the carbon emissions and identify the driving factors of the carbon emissions in the Chinese construction industry.

Some of the earlier literature has discussed the driving forces of China’s construction industry carbon emissions on a national scale [7,8,9,10]. However, it should be noted that the increase in China’s national carbon emissions are collectively shaped by the dynamics of the emissions from all provinces, municipalities and autonomous regions that comprise the country. Whether the increase in carbon emissions in the various provinces can be effectively mitigated directly affects the achievement of the national emissions reduction targets. Moreover, China’s provinces differ markedly in terms of economic development levels, industrial structures, energy consumption patterns, and many other factors. For example, while some provinces have entered the post-industrial age, others are experiencing an acceleration in the metaphase of industrialization [11]. Furthermore, there are some provinces whose industrial structures are dominated by high-tech and tertiary industries, whereas others remain heavily reliant on heavy industry [12,13]. Some provinces have gradually enhanced their levels of clean energy utilization, while others are still heavily dependent on coal consumption [14]. Hence, it is evident that a one-size-fits-all emissions reduction policy will not suffice to address the carbon emissions problems for all of China’s provinces and regions.

To address this gap, this paper separates construction industry carbon emissions into two categories, the direct category and the indirect category, to improve the accuracy of calculations based on the panel data of 30 provinces in China between 2005 and 2014. The factors that influence the construction industry carbon emissions are then analyzed and identified. The results promote a better understanding of the relationship among construction industry emissions quantities and economic development, which can contribute to developing more effective policies for each province engaged in the construction industry.

## 2. Literature Review

As China’s construction industry has played an important role in mitigating global climate change, increasingly more researchers have focused on the calculations of carbon emissions and their impact on the construction industry in China.

The literature is divided into two categories, calculation of construction industry carbon emissions and carbon decomposition analysis.

### 2.1. Calculation of Construction Industry Carbon Emissions

The construction industry is projected to contribute more than 31% of the total carbon emissions by 2020 and 52% by 2050 [1] (IPCC. Mitigation; 2011). In Europe, the construction industry accounts for over 40% of the total energy consumption [15] and contributes approximately 50% of the carbon emissions released into the atmosphere [16]. Meanwhile, the carbon emissions from construction industry in Korea comprise 23% of the country’s total carbon emissions [17], and in 2011, the UK’s construction industry-related activities accounted for an estimated 47% of its total carbon emissions [18] and 42.6 mega tons of carbon emissions [19].

To limit carbon emissions and save energy in the construction industry, a series of assessments have been established [20]. At the macro level, input-output modelling and life cycle assessments have been most commonly used [21,22]. For instance, Nassen and Holmberg [23] accessed energy use and carbon emissions in Sweden using input-output modelling, and Chen and Zhang [24] analysed the carbon emissions of China in 2007 based on a multi-scale, input-output approach [25].

However, existing studies do not focus on the characteristics of the construction industry, nor do they provide adequate insights for aggregated carbon emission policy making at the level of the construction industry. Furthermore, very limited research has been performed to estimate the construction industry carbon emissions at the provincial level in China. Considering the characteristics of the construction industry, which has close relationships with other industries, the calculation was divided into two parts, i.e., the direct carbon emissions and the indirect carbon emissions, the latter of which are from industries related to the construction industry. As our calculations are based on the data from 30 provinces in China, our results can guide future development and direct effective low-carbon policy formulation for managing carbon emissions in the construction industry at the provincial level.

### 2.2. Carbon Decomposition Analysis

Three main groups of researchers have examined the relationship among carbon emissions, energy consumption and economic growth in the literature [26]. The first group, Grossman and Krueger [27] investigated the relationship between economic growth and environmental pollution using urban area data of 42 countries. A second group of the extant literature has investigated the relationship between energy use and economic growth and presents distinct hypotheses [28,29]. For example, the unidirectional causality running from energy use to economic growth is called the growth hypothesis, which asserts that energy performs a key role in promoting economic activity and that a reduction in the energy supply will reduce economic growth [30,31]. A third group of existing studies has inspected the causal relationships among carbon emissions, energy consumption and economic growth, including Ang [32] who studied Malaysia; Jalil and Mahmud [33] and Zhang and Cheng [34] who examined China; Soytas and Sari [35] who studied the USA; Ocal et al. [36] who reported on Turkey; Chindo et al. [37] who examined Nigeria; Kuo et al. [38] who studied Hong Kong; and Albiman et al. [39] who examined Tanzania. However, their studies focused on the description of the relationship and did not reveal the combined effect of economic factors on carbon emissions.

Some studies have discussed the economy-wide assessments of the factors shaping national carbon emissions, whereas earlier studies have discussed several techniques to decompose carbon emissions. The popular decomposition methods can be divided into two groups, namely, methods linked to the Laspeyres index and methods linked to the Divisia index. One advantage of the LMDI method, which is a Divisia index method, is its ability to satisfy the factor-reversal test and the lack of unexplainable residuals in the results [40]. Based on the advantages, the LMDI has been widely applied to the study of carbon emissions at national levels. For instance, Lv et al. [41] used the LMDI to decompose the volume of historical carbon emissions in China and to analyse the country’s carbon intensity from 1980 to 2010. Li [42] used a distance function approach to decompose the change of carbon emissions in China. Gonzalez et al. [27] tracked the European Union carbon emissions through the LMDI decomposition analysis of changes in carbon emissions from 2001 to 2010. To date, there have been some attempts to use the LMDI in China’s construction industry, such as the study by Zhao et al. [43], which decomposed China’s urban residential energy consumption, Zha et al. [44], who used the Individual Development Account (IDA) to investigate the driving forces of residential carbon emissions in China, and Cai et al. [12], who decomposed China’s construction industry energy consumption. However, all of these studies focused on similar factors and found that the main factors were GDP per capita, industrial structure, population and technology level. They did not analyse the factors based on the industry characteristics.

Therefore, this study selects the LMDI as a decomposition tool to investigate the construction industry carbon emissions based on the calculation method of carbon emissions and the characteristics in the construction industry. We decompose five major factors that directly or indirectly affect the carbon emissions of construction activities, including the direct energy proportion, unit value energy consumption, value creation effect, indirect carbon intensity, and scale effect of output. These five factors illustrate the economic effects, energy effects and carbon emissions effects, which, in turn, characterize the relationship between economic development and the carbon emissions of the construction industry. This paper enriches the existing provincial characteristics analyses by synthetically considering the features of the provincial construction industry carbon emissions and their underlying driving forces. Our results and insights can be used to better deploy provincial efforts in the construction industry to abate emissions at the national level.

## 3. Research Method and Data Collection

### 3.1. Calculation of Construction Industry Carbon Emissions

In this paper, the construction industry carbon emissions are divided into two categories. The first category is for those emissions that are directly generated by the construction industry. The second category is for carbon emissions from industries related to the construction industry. These industries include the mining and washing of coal, extracting of petroleum and natural gas, mining and processing of metal ores, refining of petroleum, coking and nuclear fuel processing, manufacturing of raw chemical materials and chemical products, manufacturing of non-metallic mineral products, smelting and pressing of metals, manufacturing of metal products, as well as the transporting, storage and postal services of products.

The calculation process of direct carbon emissions of the construction industry is as follows:

(1) $$C_{D}=\sum_{i=1}E_{i}\times NCV_{i}\times A_{i}\times O_{i}\times 44/12$$

where *C_D_* denotes the direct carbon emissions of the construction industry, *i* is the type of energy, *E_i_* represents the consumption of energy *i*, *NCV_i_* represents the average lower-order calorific value of energy *i*, *A_i_* is the carbon content per unit heat of energy *i*, *O_i_* represents the oxidation rate of energy *i*, and the 44/12 is the molecular weight ratio of CO_2_ to carbon.

The calculation of indirect carbon emissions from construction-related industries is usually divided into two steps. First, we select nine industries related to the construction industry, such as mining and washing of coal; extracting of petroleum and natural gas; mining and processing of metal ores; petroleum refining; coking and nuclear fuel processing; manufacturing of raw chemical materials and chemical products; manufacturing of non-metallic mineral products; smelting and pressing of metals; manufacturing of metal products; transporting, storage and postal services of products. The equation for the direct carbon emissions of industry *j*, which is similar to Formula (2), is written as follows:

(2) $$C_{D,j}=\sum_{i=1}E_{i,j}\times NCV_{i}\times A_{i}\times O_{i}\times 44/12$$

where *C_D,j_* denotes the direct carbon emissions consumed by industry *j*, *E_i,j_* represents the use of energy *i* for industry *j*. Next, based on the input-output analysis, the indirect carbon emissions of the construction industry are calculated using the following equation [45]:

(3) $$C_{I}=\sum_{j}(C_{D,j}/IOV_{j})\times (CIOV\times y_{j})$$

where *C_D,j_* denotes direct carbon emissions of industry *j*, *j* is the category of industries, *IOV_j_* refers to the total output values of industry *j*, *CIOV* represents the construction industry output values, and *y_j_* is the total consumption coefficient of industry *j* from the construction industry, which can be derived from the input-output tables.

Finally, total carbon emissions of construction industry are as follows:

(4) $$C_{T}=C_{D}+C_{I}$$

This study then uses the construction industry carbon intensity as the dependent variable, which includes panel data of China’s 30 provinces and municipalities from 2005 to 2014 (Hong Kong, Macao, Taiwan, and Tibet are not included due to a lack of data). The construction industry carbon intensity is defined as follows in Equation (5):

(5) $$CI=C_{T}/CIOV$$

where *CI* represents the construction industry carbon intensity, *CIOV* refers to the construction industry output values, and *C_T_* denotes the total carbon dioxide emissions of the construction industry. The provincial construction industry output values are obtained from the China Statistical Yearbook.

### 3.2. The Decomposition of the Construction Industry Carbon Emissions: The LMDI Method

An index decomposition analysis, which has been widely applied to investigate the driving forces of carbon emissions, decomposes an aggregate indicator into several related driving factors and quantifies their respective contributions to the change in the aggregate indicator. Carbon emission factors can be decomposed into many elements. In this paper, these elements were reorganized into five major factors that directly or indirectly affect the carbon emissions of construction activities, including the direct energy intensity effect (*I*), the direct energy proportion (*H*), the unit value energy consumption (*F*), the value creation effect (*N*), the indirect carbon intensity (*G*), and the scale effect of output (*P*).

The carbon emissions are decomposed as in Formula (3), and corresponding notations and meanings of each factor are presented in Table 1:

(6) $$C=C_{D}+C_{I}=\frac{C_{D}}{E_{D}}\times \frac{E_{D}}{E}\times \frac{E}{S}\times \frac{S}{IG}\times IG+\frac{C_{I}}{IG}\times IG\;=I\times H\times F\times N+G\times P$$

where *E_dir_* denotes direct energy consumption of construction, *E* is the total energy consumption of construction, *IG* refers to the total output values of the construction industry, and *S* is the completed area of the construction industry, which can be derived from the China Statistical Yearbook. When carbon emissions from the construction industry are decomposed, the carbon emissions factor in the construction industry remains constant and is constant according to the calculation rules. Therefore, the direct energy intensity effect (*I*) has no effect on the construction industry carbon emissions, which this study does not continue after the calculation.

In the LMDI, the difference in carbon emissions between a target year (*t* + 1) and the datum year (*t*) is decomposed as follows:

(7) $$\Delta C=C^{t+1}-C^{t}=\Delta C_{H}+\Delta C_{F}+\Delta C_{N}+\Delta C_{G}+\Delta C_{P}$$

This study uses the multiplicative form for the decomposition as denoted in Formulas (8) to (12); the multiplicative form is as follows:

(8) $$\Delta C_{H}=\frac{C^{t+1}-C^{t}}{Ln\left(C^{t+1}-C^{t}\right)}\times Ln\left(H^{t+1}-H^{t}\right)$$

(9) $$\Delta C_{F}=\frac{C^{t+1}-C^{t}}{Ln\left(C^{t+1}-C^{t}\right)}\times Ln\left(F^{t+1}-F^{t}\right)$$

(10) $$\Delta C_{N}=\frac{C^{t+1}-C^{t}}{Ln\left(C^{t+1}-C^{t}\right)}\times Ln\left(N^{t+1}-N^{t}\right)$$

(11) $$\Delta C_{G}=\frac{C^{t+1}-C^{t}}{Ln\left(C^{t+1}-C^{t}\right)}\times Ln\left(G^{t+1}-G^{t}\right)$$

(12) $$\Delta C_{P}=\frac{C^{t+1}-C^{t}}{Ln\left(C^{t+1}-C^{t}\right)}\times Ln\left(P^{t+1}-P^{t}\right)+\frac{C_{I}^{t+1}-C^{t}}{Ln\left(C^{t+1}-C^{t}\right)}\times Ln\left(P^{t+1}-P^{t}\right)$$

## 4. Results and Discussions

### 4.1. Construction Industry Carbon Emissions Characteristics in Different Provinces

Based on the calculated direct and indirect construction industry carbon emissions of 30 provinces in China from 2005 to 2014, the detailed results and discussions are as follows (Table 2).

**Figure 1:**
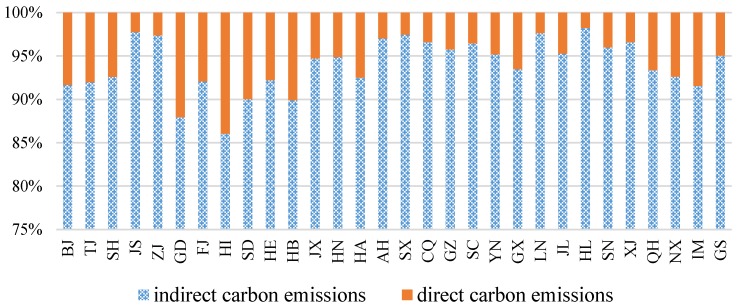
China’s provincial construction industry direct indirect carbon emissions ratio.

There are significant differences in the carbon emissions of the construction industry at the provincial level, where indirect carbon emissions of the construction industry for all provinces accounted for 90% to 95% of all carbon emissions (Figure 1). Table 3 indicates that the provinces with the highest construction industry carbon emissions were Jiangsu and Zhejiang between 2005 and 2014 and that the highest construction industry carbon emissions were approximately 500 million tons in 2014. The carbon emissions from the construction industry will increase as the development level of economic expansion increases. It is evident that the rapid economic expansion is the dominant driving force behind the acceleration of the growth of the carbon emissions of the construction industry at this stage. In contrast, Sichuan, Liaoning, Shanxi and Shandong, which are located in central China, have a low efficiency level with respect to energy utilization; therefore, the construction industry carbon emissions range between 100 and 200 million tons. Meanwhile they have relatively high construction industry carbon intensity. Specifically, the construction industry carbon emissions have fluctuated between 2005 and 2014. For example, in Shanghai, the construction industry carbon emissions peaked in 2010, reaching 81.69 million tons. The carbon emissions of the construction industry then declined by 21.5% in 2014, at 64.12 million tons. Additionally, Liaoning, Shandong, Hebei, Guangdong, Shannxi, the Inner-Mongolia Autonomous Region, Gansu and Guangxi all exhibited a similar trend as the construction industry carbon emissions were lower in 2014 than they were in 2013. The primary cause for the reductions is due to the technological progress, energy conservation and emissions reduction policies of these provinces.

**Figure 2:**
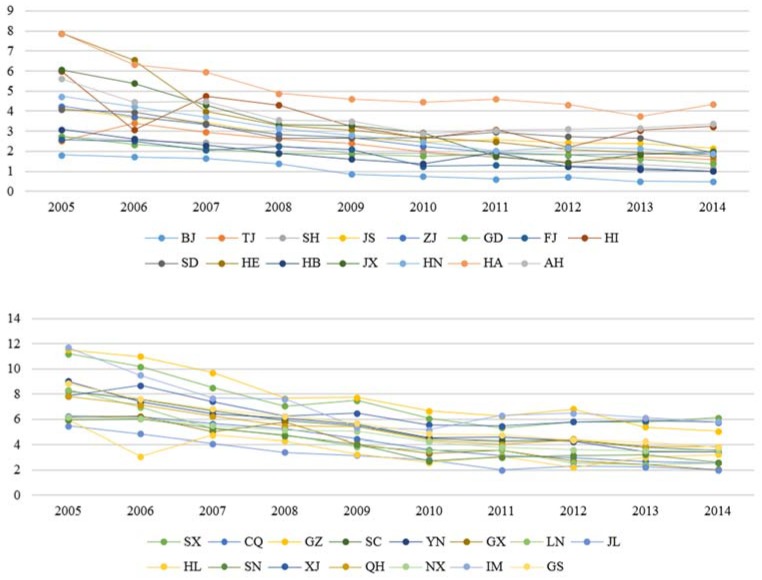
China’s provincial construction industry carbon intensity in 2005–2014.

The above identified areas of construction industry carbon emissions accounted for more than half of the total construction industry carbon emissions, and the rate of increase accounted for more than 60% compared to the other provinces. Because the northwest and central regions, including Qinghai, Hunan and Ningxia, are located inland, their technical levels and resource utilization, factors that generally result in high construction industry carbon emissions and high construction industry carbon intensity, were relatively low. These results suggest that it is necessary to control environmental problems and enhance carbon emission efficiency in these regions. In contrast, as the eastern coastal areas, such as Shanghai, Jiangsu and Zhejiang, are characterized by high levels of economic development, foreign capital utilization, population quality, energy efficiency and environmental management standards, they exhibit high construction industry carbon emissions and low construction industry carbon intensity (Figure 2). Hence, these differences should be considered when developing policies for low-carbon development.

### 4.2. The National Level Construction Industry Carbon Emissions Decomposition Results

The output scale effect factor plays a significant role in increasing construction industry carbon emissions, with a relative contribution of 35.36. In contrast, the indirect carbon intensity factor, which is the primary factor responsible for reducing construction industry carbon emissions, contributes to decreasing the growth of construction industry carbon emissions, with an integrated contribution of −27.46. The other factors, such as direct energy proportion, unit value energy consumption and value creation effect, contribute to offsetting the growth of these factors, even though the inhibitory effects of these factors are quite small, with integrated contributions of −0.11, −0.6 and −1.2, respectively.

Furthermore, the factor contributions to the construction industry carbon emissions in China dynamically change over time. From 2005 to 2014, the growth in carbon emissions was 5.99, while that of the output scale effect was 35.36. It is clear that the rapid expansion of the construction industry was the dominant force behind the acceleration of the growth of carbon emissions during this stage. In addition, the annual combined effects of these factors reveal an increasing trend during the 2005 to 2014 period. Specifically, the output scale effect demonstrates an obvious upward trend in 2008, and meanwhile, this factor’s contribution increased from 9.71 in 2007 to 24.30 in 2008. However, the construction industry carbon emissions fell due to the economic crisis, which resulted in the decline of the contribution of the output scale effect.

**Figure 3:**
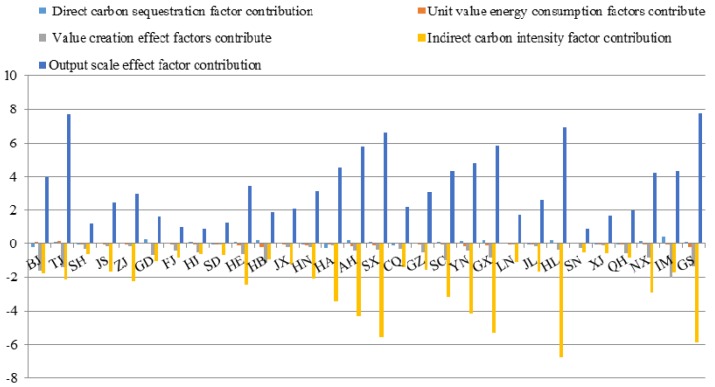
Construction industry carbon emissions factor decomposition in China.

As illustrated in Figure 3, the effects of indirect carbon intensity, which mitigate emissions, occurred during most years, and thus, they contributed to the −27.46 of the total growth. The greater inhibiting effects of carbon emissions composition of the construction industry also led to a greater reduction in carbon emissions for the period 2005 to 2014, which is the most important cause of the slowdown in the growth of carbon emissions in this factor. This suggests that an increase in indirect emissions will promote a reduction in carbon emissions under the condition of a certain construction industry output value. Additionally, during the 2005 to 2014 period, the contributions of the factors resulted in a growth trend that peaked in 2008. This peak is attributed to the global economic crisis of 2008 and the subsequent slowdown in economic development in China. Thus, the industry output value and carbon emissions of the construction industry, which is a main industry of China’s national economy, are also significantly lower. Accordingly, the contribution of indirect carbon intensity increased in 2008.

### 4.3. The Provincial Level Construction Industry Carbon Emissions Decomposition Results

#### 4.3.1. Output Scale Effect

**Figure 4:**
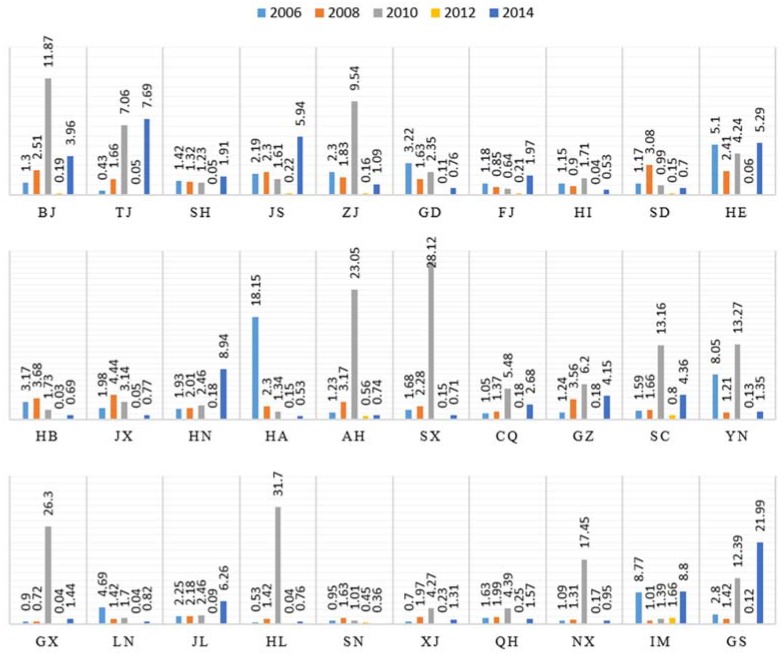
Contributions of output scale effect of 30 provinces in 2006, 2008, 2010, 2012 and 2014.

The growth of the output scale effect, which is the most important factor, is closely related to the output of construction industry. The positive effects of this factor demonstrate an obvious upward trend and relatively stable development in the provinces that characterized by high levels of economic development, energy efficiency and environmental management standards, such as Beijing, Tianjin, Zhejiang and Jiangsu, as presented in Figure 4. In contrast, this factor showed a fluctuating trend in other provinces, such as Jiangxi, Liaoning and Yunnan. This phenomenon may be affected by the national economy and provincial policies in this provinces which the construction industry development in these provinces is immature. In particular, the output scale effect has been prominent in 2010. The reason is, in order to withstand the impact of the global economic crisis in 2010, the government strongly supported the construction industry development. Therefore, the development scale of the construction industry in 2010 is relatively prominent, that is, the output scale effect effects peaks. Furthermore, the annual average contribution of the output scale effect increases between 2005 and 2014, and the contributions of this factor exhibit a growth trend that peaks in 2008, as do the national trends.

On the other hand, this factor’s contributions fluctuate from an average of 6 to 8 in the low-carbon provinces established by the state. These provinces, which include Hubei and Shaanxi, are located inland, and their technical levels and resource utilization are relatively low, factors that result in low carbon emissions efficiency. Therefore, the output of the scale effect of output of the construction industry source has a great influence in these provinces.

#### 4.3.2. Indirect Carbon Intensity

**Figure 5:**
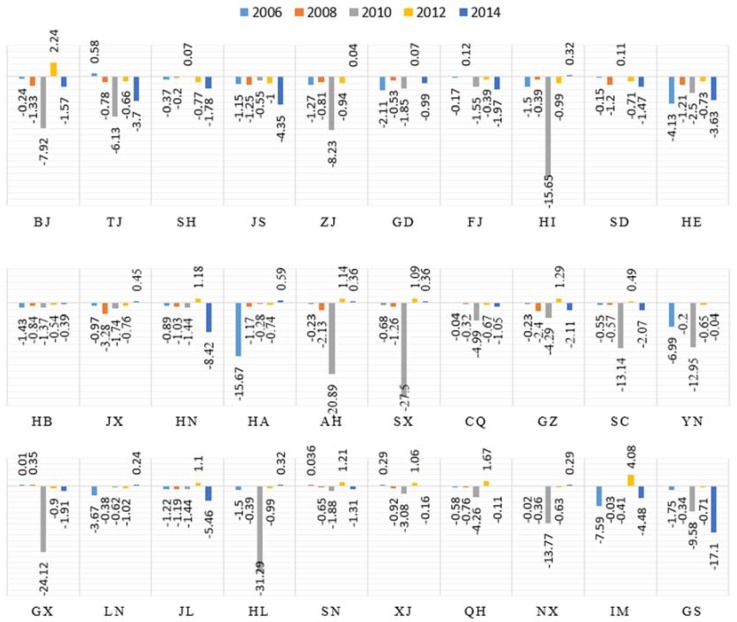
Contributions of indirect carbon intensity of 30 provinces in 2006, 2008, 2010, 2012 and 2014.

The indirect carbon intensity presents the largest rate and range of the decline in the growth of construction industry carbon emissions. As presented in Figure 5, the significant fluctuations in the contributions of indirect carbon intensity are primarily concentrated in the central, northeast and western regions, including the provinces of Shanxi, Inner-Mongolia and Gansu. These provinces, along with the low carbon provinces, exhibit low levels of technology and resource utilization, which are factors that result in low carbon emissions efficiency.

The construction industry is undergoing a qualitative change from traditional to industrialization in China in 2010. Therefore, according to the requirements of most provinces, they adjusted their industrial structure and energy structure. These modifications brought about the turning point in the contributions of indirect carbon intensity. Especially in Shanxi, Inner-Mongolia and Gansu, which located in the central and northern regions, they adjustments in construction methods have brought about drastic changes in indirect carbon intensity. We expect that indirect carbon intensity will provide strong support for carbon reduction in the provincial construction industry in the future. As the regions located in the eastern coastal areas, however, such as Guangdong and Shanghai, exhibit high levels of economic development, this factor makes a moderate contribution in these provinces.

Between 2005 and 2014, the indirect carbon intensity contributed greatly to decreasing emissions, and meanwhile, the contribution to the decrease in the fluctuations of the total carbon emissions of the construction industry was considerable during this period. Additionally, in 2010, the rate of the increase improved dramatically, but this trend was reversed in 2011. Finally, the contributions generated an annual average contribution of −20% to the total growth.

#### 4.3.3. Value Creation Effect

**Figure 6:**
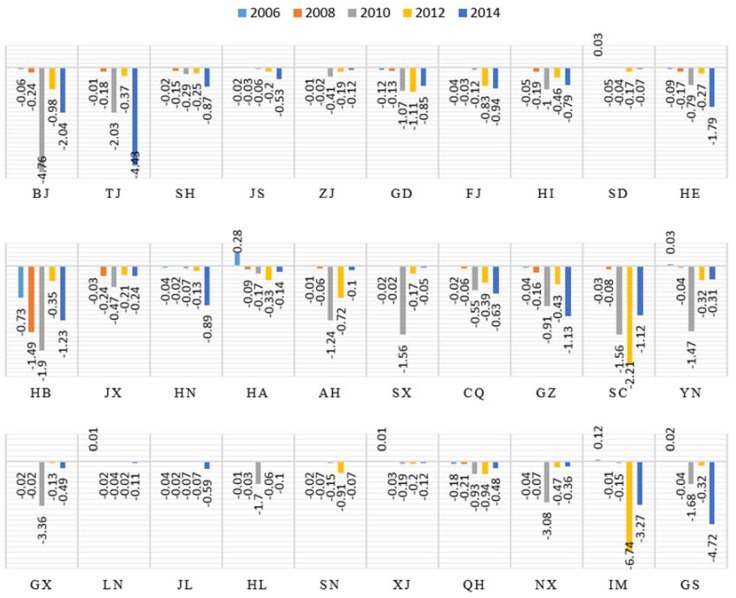
Contributions of value creation effect of 30 provinces in 2006, 2008, 2010, 2012 and 2014.

The value creation effect is second only to the indirect carbon intensity as a contributing factor to the reduction construction industry carbon emissions. As presented in Figure 6, this contribution is quite different in the eastern coastal areas, such as Shanghai, Jiangsu, Zhejiang and Guangdong, from that of the Beijing and Tianjin provinces. Furthermore, the indirect carbon intensity that contributes to the decrease in the construction industry carbon emissions assumes a greater value in the Beijing and Tianjin provinces.

The reason is that these provinces react quickly to policies regarding the economic development and environmental resources following the global economic crisis in 2010, the contributions of the value creation effect have experienced similar trends in other provinces, with the annual average integrated contributions presenting an obvious upward trend between 2005 and 2014.

As indicated in Figure 6, the contribution of the value creation effect plays a remarkable role in decreasing the carbon emissions in the northeast and western regions, such as Gansu, Inner-Mongolia, where the increments account for −4.72 of the total increment. These provinces are located inland, and their technical levels and resource utilization are relatively low, which results in a single industrial structure. In other regions, the annual average integrated contributions are approximately 1.0 out of the total increment, although fluctuating upward trend occurred between 2005 and 2014.

#### 4.3.4. Unit Value Energy Consumption

**Figure 7:**
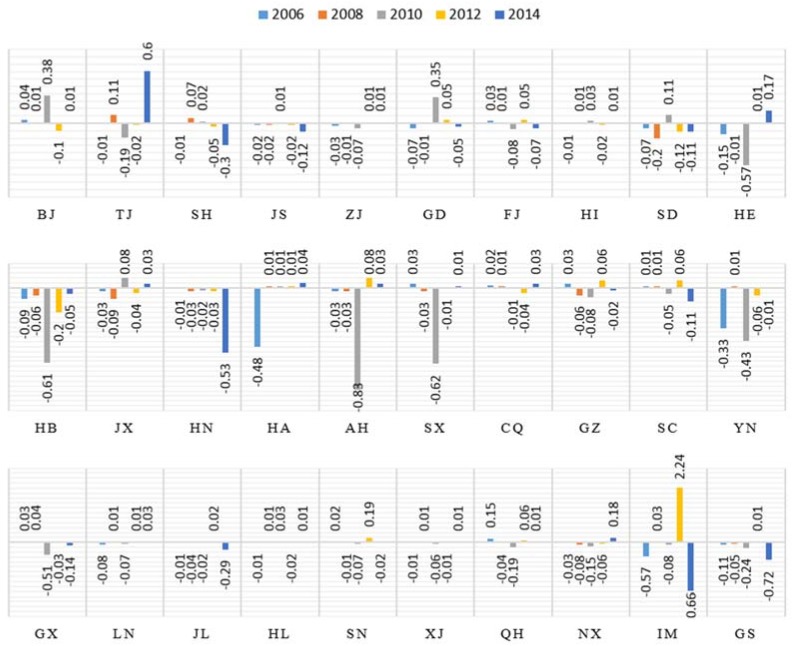
Contributions of unit value energy consumption of 30 provinces in 2006, 2008, 2010, 2012 and 2014.

Carbon emissions from the unit value energy consumption factor increased by 172.94% (the ratio of peaks and valley in the Figure 7) over the 2005 to 2014 period, which reflects the huge impact of changes in energy structure on the construction industry carbon emissions, as illustrated in Figure 7.

The contributions of the unit value energy factor with respect to the declining carbon emissions were primarily concentrated in the regions of Shanxi, Henan, and Heilongjiang, i.e., regions whose technical levels and resource utilization are relatively low. Because these provinces were experiencing rapid development, as evidenced by large construction completion areas, the contributions of the unit value energy factor was substantial. In the other regions, Chongqing, Liaoning and Xinjiang, i.e., the contributions of this factor fluctuate at −0.1.

As indicated in Figure 7, the annual combined effects of the unit value energy factor indicate an increasing trend during the 2005 to 2014 period. However, in 2010, the positive effects of the unit value energy factor changes resulted in a dramatic reduction of emissions, which is likely because the provinces began to adjust the structure of industrial construction in 2010. In general, the unit value energy factor contributes significantly to the reduction in carbon emissions and the subsequent growth trend.

#### 4.3.5. Direct Energy Proportion

**Figure 8:**
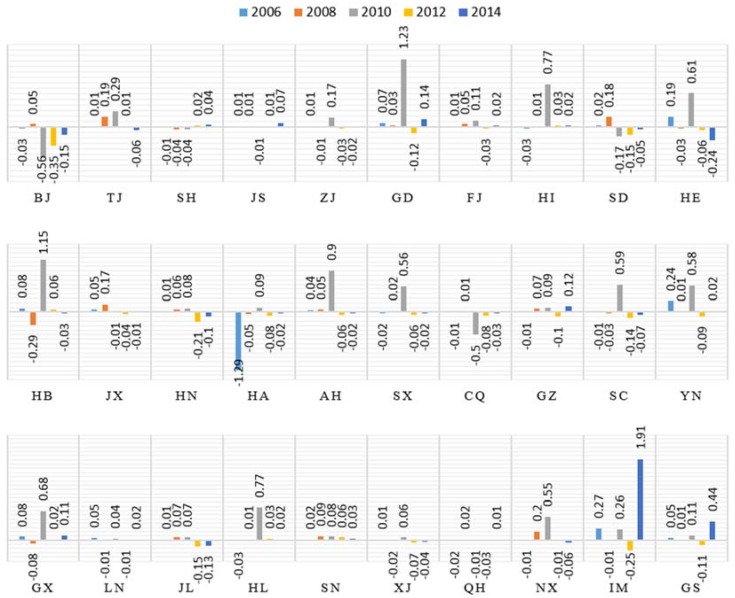
Contributions of direct energy proportion of 30 provinces in 2006, 2008, 2010, 2012 and 2014.

As indicated in Figure 8, compared with other identified factors, the direct energy proportion factor exhibits the smallest growth range regarding the reduction of carbon emissions.

Regions where the contribution value was positive are located in the northeast and western areas, which include the provinces of Heilongjiang, Gansu, Fujian, Hunan, Guangxi and Shanxi. These provinces are characterized by relatively low technical levels and low levels of resource utilization, which, in turn, result in low carbon emissions efficiency. Accordingly, as the development leads to a reduction in carbon emissions, the contribution becomes negative. The primary contributions that reduce the carbon emissions were primarily concentrated in the eastern coastal areas, where the provinces are characterized by high levels of economic development, foreign capital utilization, population quality, energy efficiency and environmental management standards. Hence, these areas served as examples to surrounding areas be demonstrating how to control environmental problems and enhance carbon emissions efficiency.

As indicated in Figure 8, this factor has a fluctuating contribution of −0.1, which indicates an increasing trend during the 2005 to 2014 period. This increasing trend is due to the effects of energy intensity and structure changes, to the development of new energy and to the reduction in on-site construction carbon emissions. Accordingly, it is concluded that the direct energy proportion is a factor the contributes to the reduction of carbon emissions.

## 5. Policy Implications

Considering the significant discrepancies across provinces, improving the accuracy of the calculations of the construction industry carbon emissions is critical for achieving China’s national action targets with respect to climate change. It is also critical to identify the contributing factors of these construction industry carbon emissions at the provincial level. Thus, the major policy proposals are summarized herein.

Carbon emissions in the construction industry in China increased by 55.6% from 2005 to 2014, with an absolute increment of 140,776 tons. Provinces such as Zhejiang, Jiangsu, and Guangdong have high construction industry carbon emissions and low carbon intensity. Other provinces, such as Shanxi, Guizhou, and Inner-Mongolia, are located inland. They exhibit high construction industry carbon emissions and low carbon emissions efficiency. These results suggest that policy makers should improve the technical knowledge and promote the coordination of economic development and environmental resources. Hence, the existing policies and measures for energy savings and emissions reduction must be steadfastly promoted to achieve a further reduction in China’s carbon intensity level, including financial incentives for energy-saving technological transformation, energy savings assessments for enterprises, and the obligatory targets for provincial-level energy consumption intensity. Meanwhile, greater effort should be made to exploit emissions reduction potential by the optimization of current energy systems. Particularly, the implementation plan and technology roadmap for realizing China’s 2050 renewable energy development goals must be detailed and executed in the near future.

The driving forces of the various provinces regarding the increase in China’s national carbon emissions changed dynamically over time. The direct carbon emissions ratio and the unit value energy consumption have fluctuating effects on carbon emissions at the national level, while the value creation effect has a negative effect on carbon emissions. Furthermore, the enhancement of indirect carbon intensity offsets the carbon emissions in all factors. Meanwhile, the output scale is the primary influencing factor with respect to the increasing emissions in China and its provinces, because the rapid expansion of construction production is the leading force behind the increase in carbon emissions. Several reasons contributed to this variation, including the level of economic development, the characteristics of the regional industrial structures, and the special policies of some provinces and cities, such as the low carbon provinces of Shaanxi, Guangxi, Hubei, Liaoning, and Yunnan.

Furthermore, the fluctuations of the various factors were more consistent between 2005 and 2014, with the peaks concentrated primarily in 2008 and 2010. In 2008, influenced by the global economic impact, the direct carbon emissions, indirect carbon emissions, completed area and total output value of the construction industry were greatly affected, which indicating the fluctuations in the various indicators. From 2010 to 2012, the change in the energy structure and the development of prefabricated construction greatly influenced the output value of the construction industry and the associated carbon proportion, thus impacting the contribution level of every index. Accordingly, the important problem regarding the future development of China is to coordinate the relationship between economic growth and carbon emissions. In addition, to reduce carbon emissions, the promotion of low-carbon building technology and the reduction of high carbon consumption with respect to building materials are essential.

## 6. Conclusions

This study separated construction industry carbon emissions into two categories, the direct category and the indirect category and adopted the LMDI decomposition method to systematically examine the contributing factors of each province. Furthermore, corresponding strategies to achieve emissions reduction advice are proposed for the different provinces based on their specific characteristics and underlying driving forces of construction industry carbon emissions. It was concluded that carbon emissions increased, whereas carbon intensity decreased dramatically, and indirect emissions accounted for 90% to 95% of the total emissions from the majority of the provinces between 2005 and 2014. The carbon intensities were high in the underdeveloped western and central regions, especially in Shanxi, Inner-Mongolia and Qinghai, whereas they were low in the well-developed eastern and southern regions, represented by Beijing, Shanghai, Zhejiang and Guangdong.

Overall, the driving forces behind the carbon emissions of the construction industry in various provinces and the contributions of these forces to the increase in China’s national construction industry carbon emissions differ greatly among each other and change dynamically over time. Moreover, it should be noted that when considering provincial differences in the driving forces of the emissions, the proposed classification of China’s provinces presented in this study varies significantly from previous studies that rely primarily on provincial geographical positions, economic developments and superficial features of carbon emissions. Therefore, the formulation of China’s emissions reduction strategies should consider both the features of provincial carbon emissions and the underlying forces shaping those features. Accordingly, these strategies must be refined in a timely manner to respond appropriately to the different developmental stages. Furthermore, the calculation and analysis methods used in this paper can also be applied to carbon emission studies in other countries. Our conclusions can also provide guidance for policies and development strategies for countries which has a massive land area and regions with greatly uneven development.

## Figures and Tables

**Table 1:** Various factors defined.

Factor Decomposition	Factors Said	Factors Explain
*C_dir_*/*E_dir_*	*I*	Direct energy intensity effect
*E_dir_*/*E*	*H*	Direct energy proportion
*E*/*S*	*F*	Unit value energy consumption
*S*/*IG*	*N*	Value creation effect
*C_ind_*/*IG*	*G*	Indirect carbon intensity
*IG*	*P*	Scale effect of output

**Table 2:** Abbreviations of the 30 study regions in China.

Region	Abbreviation	Region	Abbreviation
Beijing	BJ	Henan	HA
Tianjin	TJ	Hubei	HB
Hebei	HE	Hunan	HN
Shanxi	SX	Guangdong	GD
Inner-Mongolia	IM	Guangxi	GX
Liaoning	LN	Hainan	HI
Jilin	JL	Chongqing	CQ
Heilongjiang	HL	Sichuan	SC
Shanghai	SH	Guizhou	GZ
Jiangsu	JS	Yunnan	YN
Zhejiang	ZJ	Shaanxi	SN
Anhui	AH	Gansu	GS
Fujian	FJ	Qinghai	QH
Jiangxi	JX	Ningxia	NX
Shandong	SD	Xinjiang	XJ

**Table 3:** China’s provincial construction industry carbon emissions in 2005–2014 (Million tons).

Provinces	2005	2006	2007	2008	2009	2010	2011	2012	2013	2014	Proportion
JS	180.96	198.04	240.23	243.45	274.34	307.77	389.36	447.54	522.24	529.89	13.1
ZJ	200.35	210.64	231.76	232.39	258.00	268.11	291.22	314.23	378.97	426.67	11.0
SC	121.90	133.63	142.73	154.76	185.45	186.82	228.37	272.22	277.45	289.98	7.8
LN	123.73	123.48	110.52	119.65	130.73	166.70	223.40	194.49	210.63	200.04	6.3
SD	102.40	110.17	110.55	102.28	120.62	146.75	191.34	199.36	223.32	180.68	5.8
SX	95.03	95.76	90.17	95.27	136.84	129.95	124.00	154.70	174.82	190.63	5.1
AH	54.11	52.06	67.99	65.87	78.32	82.31	109.50	131.78	156.90	184.13	3.9
CQ	48.96	55.31	64.09	78.14	85.20	92.39	104.14	118.27	127.52	141.71	3.6
HE	101.31	94.70	64.27	67.36	77.40	86.86	97.98	101.82	100.94	98.40	3.5
GD	60.34	60.60	63.95	62.16	71.20	82.47	106.93	119.93	127.04	113.78	3.4
SN	39.12	49.99	62.34	77.93	93.41	77.77	106.30	111.45	127.91	119.37	3.4
YN	48.71	49.56	48.53	55.02	67.13	68.60	85.84	101.64	101.15	106.02	2.9
HB	41.73	43.69	48.66	49.97	54.50	60.53	109.37	87.43	90.86	99.13	2.7
SH	51.56	58.27	61.52	73.93	72.26	81.69	74.31	69.48	69.05	64.12	2.7
IM	44.55	44.43	52.43	59.40	51.54	58.22	88.00	93.78	96.38	81.36	2.6
XJ	28.39	33.50	33.57	39.21	51.44	53.82	72.70	94.37	123.32	134.23	2.6
HL	34.32	21.50	41.61	44.73	43.70	46.65	62.65	52.64	75.44	69.61	1.9
GZ	31.23	34.37	33.93	30.20	40.61	41.43	51.74	70.68	74.23	82.69	1.9
JX	34.22	36.05	33.87	34.47	43.45	49.09	36.39	39.94	64.13	82.08	1.8
FJ	23.06	29.77	33.05	42.86	48.25	36.81	48.20	56.20	63.91	67.83	1.8
TJ	18.85	33.54	36.13	37.84	45.82	47.65	51.10	47.95	62.99	65.51	1.8
GS	27.77	26.21	29.72	30.08	33.21	36.18	43.87	59.91	73.06	69.45	1.7
GX	26.00	32.13	30.76	43.35	38.08	40.58	55.34	50.89	55.72	53.23	1.7
HA	18.94	19.89	26.59	31.70	38.38	44.78	47.65	48.24	53.78	67.64	1.6
BJ	34.43	37.37	42.57	41.97	35.13	38.33	38.14	47.32	37.17	38.46	1.5
JL	26.64	29.30	30.35	34.01	36.33	37.81	32.59	45.80	49.78	50.22	1.5
HN	22.99	25.74	27.39	31.10	32.11	33.42	32.97	43.59	47.04	47.01	1.3
NX	6.99	7.93	8.52	9.84	13.11	14.51	16.38	17.01	20.01	22.73	0.5
QH	7.11	7.74	7.86	7.80	11.03	12.08	12.93	14.34	16.05	16.65	0.4
HI	2.28	2.40	2.53	3.66	3.86	4.20	5.72	5.51	4.98	4.07	0.2
